# From toddler to school age: longitudinal motor and cognitive outcomes in children with critical congenital heart disease

**DOI:** 10.3389/fped.2026.1943055

**Published:** 2026-09-23

**Authors:** Eveline Esther Oppelaar, Maaike Sprong, Monique van Schooneveld, Nicolaas J. G. Jansen, Selma O. Algra, Renske Schappin, Johannes M. P. J. Breur

**Affiliations:** 1 Department of Dermatology, ErasmusMC, Rotterdam, Netherlands; 2 Center of Child Development, Exercise and Physical Literacy, Wilhelmina Children’s Hospital, University Medical Center Utrecht, Netherlands; 3 Department of Medical Psychology, Wilhelmina Children’s Hospital, University Medical Center Utrecht, Netherlands; 4 Department of Pediatric Intensive Care, Wilhelmina Children’s Hospital, University Medical Center Utrecht, Netherlands; 5 Department of Radiology & Nuclear Medicine, Division Imaging & Oncology, University Medical Center Utrecht, Netherlands; 6 Department of Medical Psychology, Wilhelmina Children's Hospital, University Medical Center Utrecht, Netherlands; 7 Department of Pediatric Cardiology, University Medical Center Utrecht, Utrecht, Netherlands

**Keywords:** cognitive development, critical congenital heart disease, executive function, motor development, neurodevelopment, quality of life

## Abstract

**Background:**

Despite improved survival, children with critical congenital heart disease (CCHD) face motor, cognitive, and executive functioning (EF) deficits. This can negatively impact their quality of life, yet longitudinal data on these domains and their interrelationships, remain scarce.

**Methods:**

In this longitudinal cohort study, 29 children with CCHD were assessed at 2 years, of whom 27 children were reassessed at 5 years, and 25 children at 8 years. Age-appropriate standardized instruments were used. Associations were examined using Spearman correlations, with Bonferroni-Holm correction for multiple comparisons.

**Results:**

Large correlations emerged between the cognition score at 2 years and the total intelligence quotient (IQ) score at 8 years (*r* = .70, *p* < .001), and between the total IQ score at 5 years and the total IQ score at 8 years (*r* = 0.82, *p* < .001). Cognition scores did not differ from normative means. No significant correlations were found between the motor outcomes at the 3 timepoints. The total motor score at age 5 (M = 6.6, SD = 2.5), and at age 8 (M = 43.6, SD = 7.6) were significantly lower compared to normative means. A substantial proportion of children scored below the 25th percentile on EFs auditory attention (42%; M = 10.2, SD = 3.5) and shifting (35%; M = 11.2, SD = 2.6). Longitudinal cluster analyses revealed a steady low and steady high cluster in both motor and cognitive development with lower birth weight as a risk factor for cognitive impairments.

**Conclusion:**

Two subgroups emerged with a relatively low, and a relatively high motor and cognitive performance group despite fluctuations in absolute test scores, with lower birthweight forming a risk factor for cognitive impairments. Overall, children showed impaired motor development compared to the normative means. Larger studies are needed to examine the interplay between motor, cognition, and EF development.

## Introduction

Congenital heart disease (CHD) occurs in approximately 9 per 1,000 live births (1). Around one-fourth of all CHDs require open heart surgery using cardiopulmonary bypass in the neonatal period, classifying them as critical congenital heart disease (CCHD) (2). Over the past few decades, survival rates for children with CHD have significantly improved, with more than 90% surviving into adulthood (3). With increased survival, there has been a growing recognition for the risk of neurodevelopmental problems in these children, caused by a number of factors such as genetic predisposition, placental pathology, other environmental and maternal influences, hypoxia, hypoperfusion, altered brain maturation, as well as perioperative brain injury (4–7). Specific problems are motor and cognitive impairments and executive functioning (EF) deficits as children grow older and their developmental demands surpass their EF abilities (growing into deficit) (8–10). Cognition refers to an individual's capacity for learning, reasoning, and problem-solving (11). EF are higher-order cognitive functions, including attention, working memory, inhibition, and cognitive flexibility (10). EF and general cognition are related but distinct neurodevelopmental domains with cognition typically measured with standardized cognitive composite and intelligence quotient (IQ) measures, and EF through separate instruments targeting its distinct functions (12). Impairments in EF, along with motor and cognitive developmental problems, can negatively impact physical functioning, academic functioning, social well-being, and health-related quality of life (12, 13). Research suggests that motor, cognitive, and EF development are interconnected in intricate, bidirectional relationships, likely mediated in part by shared neural networks (14). Therefore, longitudinal studies on the evolution of and the interplay between these neurodevelopmental domains are essential. Yet only limited studies in children with CCHD presented longitudinal data on motor or cognitive development across early childhood. Longitudinal studies beyond toddler and preschool age remain scarce, and the interplay between motor, cognitive, and EF development in children with CCHD is poorly understood (15–19).

Consequently, this study aims to describe the longitudinal motor and cognitive developmental outcomes in CCHD after neonatal cardiac surgery at 2 years (toddler), 5 years (preschool), and 8 years (school age). Secondary, we aim to explore the potential interplay between motor, cognitive, and EF development, and patient characteristics. This knowledge will contribute to better prediction and early identification of significant developmental deficits, which hold important value in the clinical assessment and treatment of children with CCHD.

## Materials and methods

### Study sample

The original study population consisted of 37 children with an aortic arch obstruction requiring neonatal single ventricle or biventricular repair with cardiopulmonary bypass (see Figure 1) (4). These children were part of a randomised controlled trial on the incidence of perioperative cerebral injury after specific perfusion techniques. The trial was performed between 2009 and 2011 in the Wilhelmina Children's Hospital, Utrecht, the Netherlands. Inclusion criteria were all neonates requiring aortic arch reconstruction between January 2009 and May 2012. Exclusion criteria were age at surgery >4 months, high suspicion of or confirmed of a genetic syndrome, the need for sedation and intubation exclusively for preoperative MRI, or an expected aortic arch reconstruction time exceeding 60 min. No exclusion criteria were applied for perioperative neurological complications such as stroke or intraventricular haemorrhage. All surviving patients from the original trial were eligible for neurodevelopmental follow-up, and were re-approached for each assessment by research staff at each timepoint. The 8 year assessment was added subsequently as more funds became available. The Medical Review Board of the University Medical Center Utrecht approved the study, and parental informed consent was obtained for use of clinical data for research purposes (No. 17-003). All information except for developmental test data was extracted from the medical records. Patients and their parents were not involved in the design, conduct, analysis, or reporting of this study. We will enlist the help of the cardiac patient organisation in disseminating the results. We adhered to the STROBE reporting guideline (see Supplementary S1 for the reporting checklist).

**Figure 1:**
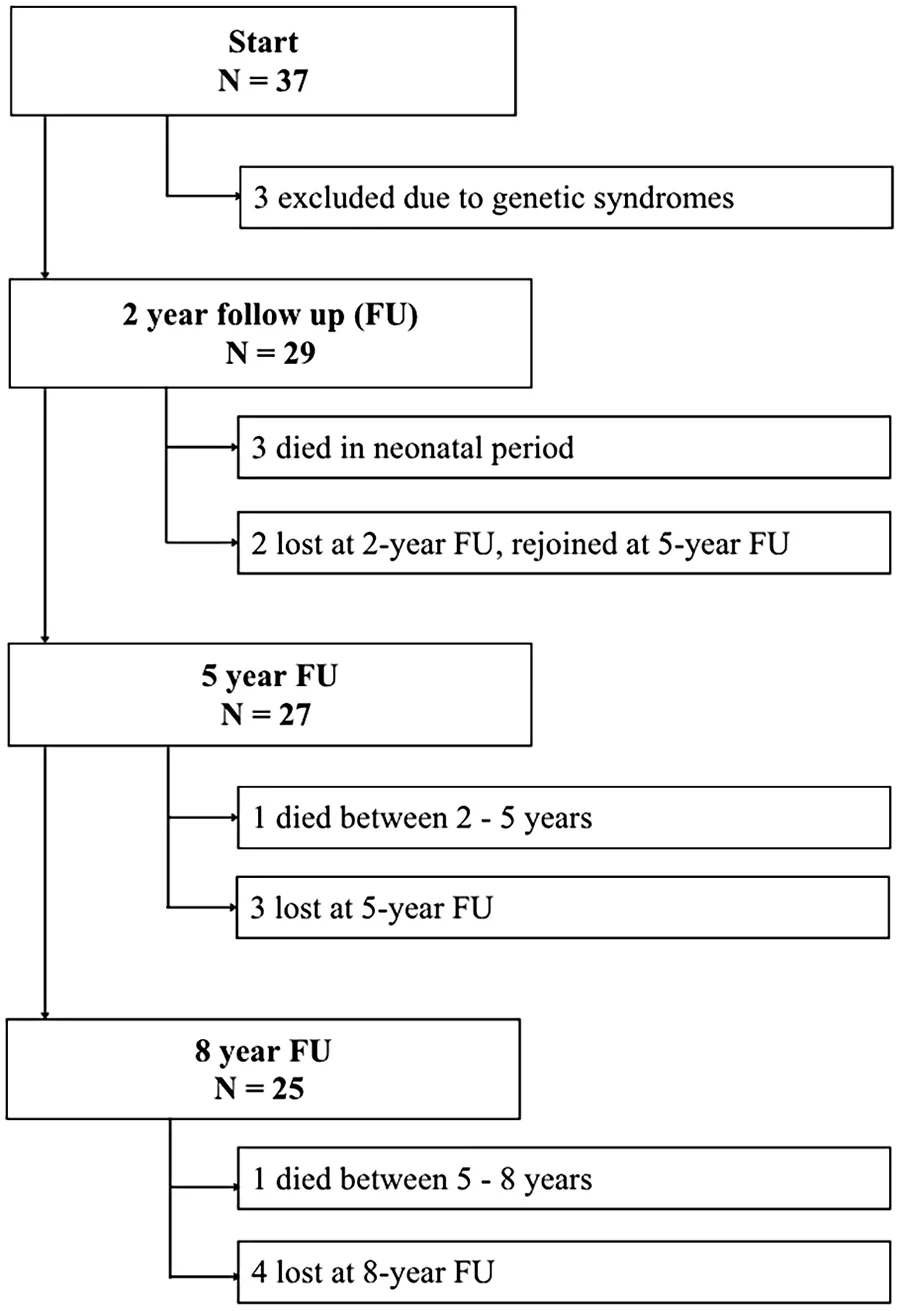
Participant flow.

### Outcome assessments

#### Motor outcome

Motor outcomes at 2, 5, and 8 years were assessed with three standardized age-appropriate measurements (see Supplementary S2 for a detailed description). At 2 years, the fine and gross motor scaled scores (mean 10, SD 3), and the total motor composite standard score (mean 100, SD 15) were used of the Dutch version of the Bayley Scales of Infant Development, Third Edition (Bayley-III-NL) (20).

At 5 years, the Movement Assessment Battery for Children—Second Edition (Movement ABC-II-NL) was used (21). The Movement ABC-II-NL is a global test of motor competence, with assessments of fine and gross motor coordination. The total motor standard score, and three domain standard scores (manual dexterity, aiming & catching, balance) were used (mean 10, SD 3).

At 8 years the Bruininks–Oseretsky Test of Motor Proficiency, second edition (BOT-II-NL), was used (22). This screening tool for children aged 4–21 years, measures gross motor and fine motor skills. The subtests (fine motor precision, fine motor integration, manual dexterity, upper-limb coordination, bilateral coordination, balance, running speed & agility, strength) aggregate into 5 motor domain composite scores (fine manual control, manual coordination, body coordination, strength & agility, total motor). The subtest scaled scores (mean 15, SD 5) and motor domain composite scores were used (mean 50, SD 10). The BOT-II-NL was selected because it provides a broader domain coverage than the MABC-2-NL, which comprises only three components (22, 23).

#### Cognitive outcome measures

Cognitive outcomes at 2, 5, and 8 years were assessed with three standardized age-appropriate instruments (see Supplementary S2). At 2 years, the cognitive composite score of the Bayley-III-NL was used (mean 100, SD 15) (20). At 5 years, the Dutch Wechsler Preschool and Primary Scale of Intelligence (WPPSI-III-NL) was used (24). The total intelligence quotient (TIQ) was used (mean 100, SD 15). The processing speed index (PS) was also used, as executive functions are involved in the associated subtests (21).

At 8 years, the WISC-III-NL (25) or the WISC-V-NL (26) was used. The WISC-V-NL was used for the last 4 participants when this new version of the test became available near the end of the study. The TIQ and PS of these instruments were used (mean 100, SD 15). At 8 years of age, the cognitive measure was administered together with the executive functioning measure.

For motor and cognitive outcomes, scores ≤-1SD (16th percentile) below the normative mean were classified as below average, and scores ≤-2SD (2.5th percentile) as indicating significant delay, applied consistently regardless of instrument-specific labelling conventions.

#### Executive functioning measure

Executive functioning (EF) at 8 years was measured with subtests of the Developmental Neuropsychology Assessment, 2nd edition (NEPSY-II-NL) for children 3:0–12:0 years of age (27). We initially administered a wider set of preselected subtests across multiple domains to obtain a broader neuropsychological profile while limiting assessment burden. Based on the secondary aim of our study to assess EF, and the level of missing data across this broader battery, only subtests within the attention and EF domain (AARS and Inhibition) are reported. These tasks include a switching condition, which allows the assessment of attention, inhibition, and cognitive flexibility (Korkman 2007). A composite score was derived for participants completing at least 2 of the 4 subtests. Unlike the motor and cognitive outcomes, Dutch scaled norms are unavailable for the EF tasks and SD-based classifications therefore cannot be obtained. Instead, American normative data was used, and Dutch percentile scores were reported to identify children scoring below average. Scores above the 25th percentile reflect average EF performance, and scores below the 25th percentile indicate lower EF performance, encompassing the low average and below average ranges defined for the other outcome domains.

### Statistical analysis

Descriptive statistics were calculated as frequencies, percentages (%), mean and SD, or median [interquartile range (IQR)] depending on the type and distribution of data. To compare scores obtained from different tests, outcomes were transformed into z-scores. Associations between motor, cognitive, and EF outcomes at different ages were examined using Spearman correlations, with *post-hoc* Bonferroni-Holm correction for multiple comparisons. Given the large number of correlations and the small sample size, only statistically significant correlations after Bonferroni-Holm correction are reported. Non-significant correlations are available from the corresponding author upon request. We interpreted the found associations according to the criteria of Cohen's *d* for a small effect (*d* = 0.2), medium effect (*d* = 0.5) and large effect (*d* = 0.8) (28). To identify trajectories of motor and cognitive development, k-means longitudinal clustering on the total motor respectively cognitive outcomes at age 2, 5, and 8 was performed in R using the “kml” package (29). Participants with outcomes missing at more than one timepoint were excluded, and remaining missing data was imputed using the copy mean method. The optimal number of clusters was based on 20 iterations and determined using the Calinski/Harabasz criterion (see Supplementary S3). Differences in descriptive statistics for separate clusters were investigated using *t*-test or the non-parametric equivalent. All statistical analyses except k-means clustering, were performed with IBM SPSS® version 25.0 (SPSS, Chicago, IL). A *p*-value < 0.05 was considered significant.

## Results

### Patient characteristics

Patient characteristics are presented in Table 1.

**Table 1:** Baseline characteristics of the study cohort.

Characteristic	*n* = 31^a^
Male, *n* (%)	26 (84%)
Birth weight, median *grams (IQR)*	3,350 (521.3)
Gestational age, median *weeks (IQR)* (*n* = 30)	39.2 (1.3)
Balloon Atrio Septostomy (BAS) *n* (%)	1 (3)
Age at surgery, mean days (SD) *(IQR)* (*n* = 24)	11.0 (8–12)
Congenital heart disease, *n* (%)	
Single Ventricle pathology	10 (32%)
- Hypoplastic left heart	6 (19%)
- Hypoplastic right heart	4 (13%)
Biventricular pathology	21 (68%)
- Hypoplastic left heart complex	5 (16%)
- Taussig Bing anomaly	4 (13%)
- Aortic arch obstruction	12 (39%)
Primary surgical procedure, *n* (%) (*n* = 24)	
- Norwood procedure	10 (42%)
- Arterial switch operation with aortic arch repair	1 (4%)
- Arterial switch operation with aortic arch repair and VSD closure	1 (4%)
- Aortic arch repair	7 (29%)
- Aortic arch repair with VSD closure	5 (21%)
Maternal education level^b^	
Low—Middle	15 (48%)
High	14 (45%)
Unknown	2 (7%)

a Data are presented for the 31 surviving patients without genetic syndromes. The 3 deceased patients and 3 patients with a genetic syndrome were excluded from the analyses.

b According *to the ISCED classification system.*

#### Additional therapeutic support

During follow-up, 16 children at 2 years (52%), 19 children at 5 years (61%), and 9 children at 8 years (29%) received additional therapeutic support. This consisted mainly of physiotherapy (45% at 2 years, 23% at 5 years, 13% at 8 years), psychological support (0% at 2 years, 19% at 5 years, 7% at 8 years), and others (mainly speech therapy; 26% at 2 years, 42% at 5 years, 16% at 8 years). These categories were not mutually exclusive as some children received multiple forms of support.

### Outcome

#### Motor outcome

As displayed in Table 2, children's motor development at 2 years showed a mean motor composite score of 102.9 (10.0), with no children exhibiting below average results, vs. 9% in the normative population.

**Table 2:** Motor development outcome at 2, 5 and 8 years compared with test norms.

2 years follow up (Bayley-III; *n* = 29)	M (SD)	Norm −1SD < x ≤ −1.5SD, *n* (%)	Norm ≤ −1.5SD, *n* (%)
Age at test (months)	24.5 (0.8)		
Total motor composite score	102.9 (10.0)	2 (7)	0 (0)
Fine Motor Scaled Score (FMSS)	12.3 (2.5)*	0 (0)	1 (3)
Gross Motor Scaled Score (GMSS)	8.3 (2.2)*	4 (14)	3 (10)

5 years follow up (M-ABC-II; *n* = 23)	M (SD)	Norm −1SD < x ≤ −1.5SD, *n* (%)	Norm ≤ −1.5SD, *n* (%)
Age at test (years)	5.7 (0.3)		
Total motor score	6.6 (2.5)*	5 (22)	10 (44)
Domain Standard Scores			
Manual Dexterity	7.0 (2.8)*	4 (17)	9 (39)
Ball Skills	7.2 (2.9)*	1 (4)	9 (39)
Balance	7.7 (2.8)*	9 (39)	3 (13)

8 Years Follow up (BOT-II; *n* = 24)	M (SD)/Median [IQR]	Norm −1SD < x ≤ −1.5SD, *n* (%)	Norm ≤ −1.5SD, *n* (%)
Age at test (years)	8.5 (0.5)		
Total motor composite SS	**43.6** (7.6)*	**7** (**29)**	**3** (**13)**
Fine Motor Precision (FMP)	11.7 (4.3)*	9 (38)	3 (13)
Fine Motor Integration (FMI)	12.4 (4.7)	6 (25)	4 (17)
Fine Manual Control (FMC) SS	**44.0** (8.7)*	**5** (**21)**	**4** (**17)**
Manual Dexterity (MD)^a^	12.5 [9 −16]*	3 (13)	0 (0)
Upper-Limb Coordination (ULC)	11.4 (3.5)*	4 (17)	4 (17)
Manual Coordination (MC) SS	**42.5** (5.7)*	**7** (**29)**	**2** (**8)**
Bilateral Coordination (BC)	13.3 (3.7)	5 (21)	0 (0)
Balance (B)	12.7 (5.2)	4 (17)	4 (17)
Body Coordination (BCSS) SS	**44.8** (8.9)*	**4** (**17)**	**3** (**13)**
Running Speed & Agility (RSA)	14.2 (3.9)	2 (8)	1 (4)
Strength (S)	14.7 (4.6)	1 (4)	3 (13)
Strength and Agility (SA) SS	**48.5** (**9.5)**	**4** (**17)**	**1** (**4)**

Note. Normative means and standard deviations are as follows: Bayley-III-NL motor composite score is reported as a *standard score* (M = 100, SD = 15); domain scores as *scaled scores* (M = 10, SD = 3). M-ABC-II-NL total score and domain scores are reported as *standard scores* (M = 10, SD = 3). BOT-II-NL subtest *scaled scores* are normed as M = 15, SD = 5; the 5 motor domain *composite scores* are normed as M = 50, SD = 10. Data presented as mean (SD), median [IQR] depending on distribution and *n* (%). BOT-II-NL subtest scaled scores are normed as M = 15, SD = 5; the 5 motor domain composite scores (bold) are normed as M = 50, SD = 10.

a Data are not normally distributed*.*

* *p* < 0.01 vs. norm group mean.

By 5 years, the mean motor development score was 6.6(2.5), and 10 children (44%) scored ≤ −1.5SD, (expected 9%), indicating below average motor performance. Difficulties were most commonly observed in manual dexterity (9 children; 39%), followed by aiming and catching (9 children; 39%) and balance (3 children; 13%).

At 8 years, motor assessments showed a mean motor composite score of 43.6(7.6), with 3 children (13%) performing ≤ −1.5SD vs. 9% in the normative population.

#### Motor outcome at 8 years related to early motor outcomes

No significant relations were found between motor development at 2 years and 5 years, or between 2 and 8 years. The expected significant intercorrelations among subscales within the same instrument were not reported to avoid redundancy (see Supplementary S4 for the correlation matrix).

#### Cognitive outcome

At 2 years, the mean cognitive composite score was 103.5 (16.9) as displayed in Table 3 with no children exhibiting below average results, vs. 9% in the normative population. At 5 years, the mean total intelligence quotient (TIQ) was 94.1. [83.5–107.0] with 2 children (10%) scoring below average (≤1.5SD, expected 9%). Additionally, 8 children (35%) scored below average on processing speed (≤1.5SD) vs. 9% in the normative population. At 8 years, the mean TIQ was 96.5 (15.5) with 3 children (14%) scoring below average on total IQ (≤ 1.5SD) vs.9% in the normative population, and 1 child (5%) scoring below average on processing speed (≤1.5SD, expected 9%).

**Table 3:** Cognitive development outcomes compared with test norms.

2 years follow up (Bayley-III-NL)	M (SD)	Norm −1.5SD < x ≤ −1SD, *n* (%)	Norm ≤ −1.5SD, *n* (%)
Age at test (months)	24.5 (0.8)		
Total cognitive composite score (*n* = 29)	103.5 (16.9)	3 (10)	0 (0)

5 years follow-up (WPPSI-III-NL)	M (SD)/Median [IQR]		Norm ≤ −1.5SD, *n* (%)
Age at test (years)	5.6 (0.5)		
Processing Speed score (*n* = 23)	84.8 (13.1)*	5 (24)	8 (35)
Total IQ (*n* = 21)^a^	94.1 [83.5–107.0]	5 (24)	2 (10)

8 years follow-up (WISC-III-NL, WISC-V-NL)	M (SD)		Norm ≤ −1.5SD, *n* (%)
Age at test (years)	8.5 (0.6)		
Processing Speed score (*n* = 22)	99.2 (13.6)	3 (14)	1 (5)
Total IQ (*n* = 23)	96.5 (15.5)	3 (14)	3 (14)

Note. Normative means and standard deviations are as follows: Bayley-III-NL cognitive composite score is reported as a *standard score* (M = 100, SD = 15). WPPSI-III-NL and WISC-V-NL IQ scores and processing speed scores are reported as *standard scores* (M = 100, SD = 15). Data presented as mean(SD), median [IQR] depending on distribution and *n* (%).

a Data are not normally distributed.

* *p* < 0.01 vs. norm group mean.

#### Cognitive outcomes at 8 years related to earlier cognitive outcomes

A large significant correlation was found between the cognition score at 2 years with the total IQ score at 8 years (*r* = .71, *p* < .001), and between total IQ score at 5 years with the total IQ score at 8 years (0.82, *p* < .001). The expected significant intercorrelations among subscales within the same instrumentnot reported to avoid redundancy (see Supplementary S4 for the correlation matrix).

#### Executive functioning at school age

Children were assessed with subtests of the neuropsychological test battery NEPSY-II-NL to measure their executive functioning. More children than the normative population scored below average on the auditory attention task, as displayed in Table 4.

**Table 4:** Executive functioning outcome at 8 years compared with test norms.

NEPSY-II-NL Subtest	M (SD)/Median [IQR]	*N* (%) ≤ 25	26 ≤ *N* (%) ≤ 75	76 ≤ *N* (%)
Auditory attention total correct (*N* = 12)	10.2 (3.5)	5 (42)	7 (58)	0 (0)
Auditory attention—shifting response set (*N* = 11)	11.2 (2.6)	4 (36)	7 (64)	0 (0)
Inhibition total incorrect (*N* = 12)^a^	15.9 [14.3–18.8]	0 (0)	12 (100)	0 (0)
Inhibition—switching total time needed (*N* = 12)^a^	10.8 [8.3–13.0]	1 (8)	10 (83)	1 (8)
Composite score (*N* = 15)	12.0 (3.2)			

Note. Percentile scores at or below 25 represent scores equal to or lower than 25% of the scores and can be seen as below average. Data presented as mean (SD) or median [IQR] depending on distribution.

a Data are not normally distributed.

#### Explorative analysis: interplay between motor, cognitive and EF development

No significant correlations were found between motor and cognitive development, nor between these domains and EF.

#### Explorative longitudinal cluster analysis

The application of k-means longitudinal clustering to the total motor outcome and cognitive outcome over time identified two clusters in both motor and cognitive outcomes, as shown in Figure 2.

**Figure 2:**
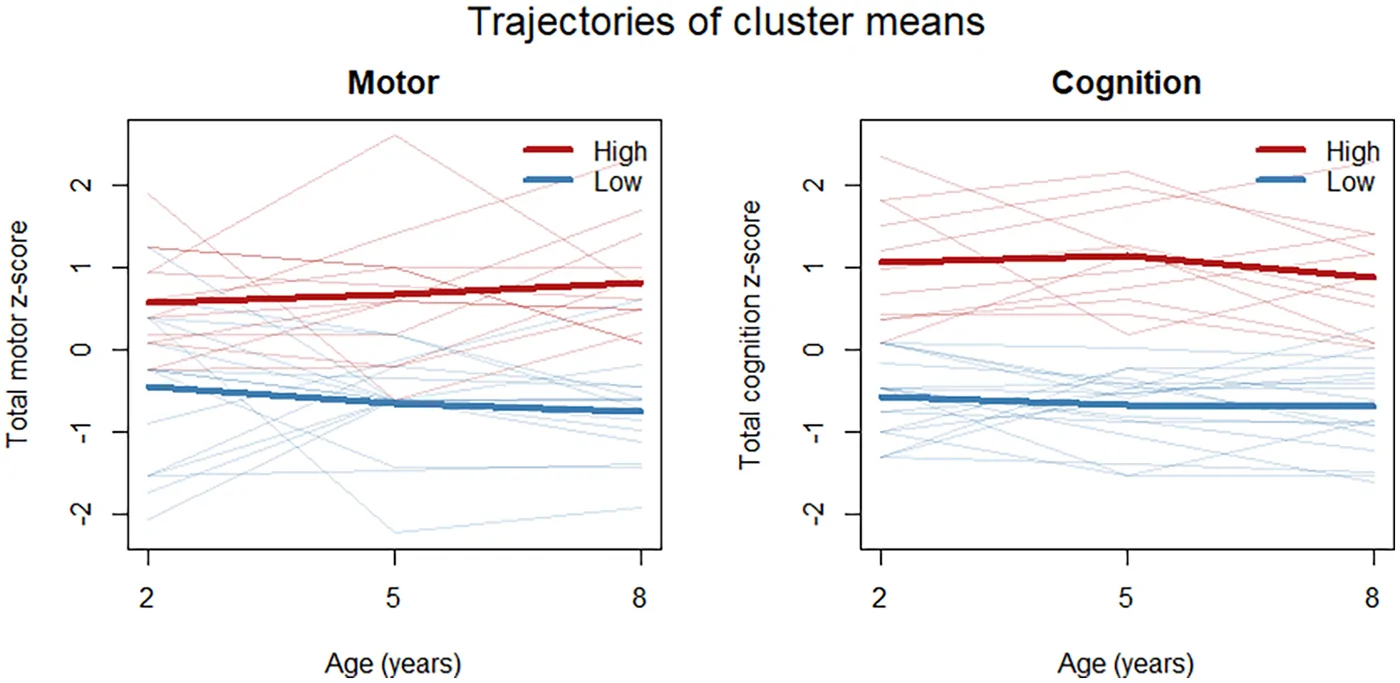
Clusters in motor outcomes (*N* = 29) and cognition outcomes (*N* = 29) over time.

The high motor cluster (45%of participants) is characterized by a relatively stable motor outcome across all 3 time points of around +0.7 SD above the total motor score (see Supplementary S5). Cluster 2 (55% of participants) also shows a stable motor outcome across all 3 timepoints of approximately −0.6 SD. Two clusters also emerge in the total cognitive outcomes over time, as shown in Figure 2. The high cognition cluster (37% of participants) shows a stable cognitive outcome across all 3 timepoints of around +1 SD, corresponding to a total IQ score of approximately 115. The low cognition cluster 63%) is characterized by a relatively stable cognitive outcome across all timepoints of around −0.6 SD above the total cognition score, corresponding to a total IQ score of approximately 91 (see Supplementary S3).

No significant differences between clusters were observed for gender, CHD diagnosis, primary surgical procedure, gestational age, operation age, or maternal educational level. Significant differences were found in birthweight between the cognitive clusters (Mann–Whitney *U* = 36.0, *p* = .004), with lower birthweight related to worse cognitive functioning.

## Discussion

This study aimed to describe longitudinal motor and cognitive outcomes in children with critical CHD (CCHD) after neonatal cardiac surgery at 2 years (toddler), 5 years (preschool), and 8 years (school age). It also explored potential interactions between motor development, cognition, and executive functioning (EF) at school age. Overall, children with CCHD scored lower on motor measures compared to normative data,. Most children scored comparable to the norm on cognitive measures. Cognitive performance at 2 years and at 5 years was associated with cognition at school age. Despite fluctuations in absolute test scores, we observed a relatively stable high performance group and low performance group over time within each domain. with birth weight emerging as a risk factor for cognitive impairments.

Motor functioning appears to fluctuate in test performance, especially between ages 2 and 5 years. This may be partially explained by differences in how motor competence is operationalized across instruments. The Bayley-III-NL assesses whether a child attains motor milestones. The Movement ABC-II-NL evaluates speed, accuracy, and precision with which the motor tasks are executed. Tasks demanding fast, accurate, or sustained performance draw more heavily on higher-order cognitive functions than milestone attainment does (30). Therefore, these instruments reflect related but distinct constructs, and this shift in what is being demanded of the child limits direct comparison across timepoints. The Movement ABC-II-NL and the BOT-II-NL do both measure motor proficiency, but differ in structure and item content (23). These differences are in line with the absence of a significant relation between motor outcomes at the three timepoints in our study.

Separately from this discontinuity between instruments, mean motor scores were significantly lower than normative means at 5 and 8 years. A substantial proportion of children scored in the low average range at both timepoints, which is broadly consistent with reported ranges in the wider CHD literature at comparable ages (31, 32). Scores in the low average range do not, on their own, indicate clinical impairment. However, the significantly lower mean scores compared to the normative population indicate a meaningful degree of motor difficulty at group level, and the substantial proportion scoring ≤1.5SD indicates a meaningful subgroup performing outside of the expected range.

Our findings may partly reflect the heart defect risk profile of our cohort, as CHD severity ranged from single ventricle physiology which has been associated with more severe motor impairment (31), to a range of biventricular defects of varying complexity. Our sample was underpowered to formally test the association between CHD severity and motor outcomes. However, prevention or intervening on motor problems is important as fine motor difficulties are associated with educational problems (33), especially in combination with cognitive impairments. Furthermore, gross motor difficulties can complicate participation in exercise and sports and result in a sedentary lifestyle and social isolation from peers (34).

In contrast to motor functioning, the cognition results showed significant correlations between 2 and 8 years, and between 5 and 8 years. The Bayley-III-NL cognitive composite score reflects broad developmental attainment, whereas the WPPSI-III-NL and WISC-III/V-NL total IQ scores reflect a more differentiated profile of cognitive abilities and draw more heavily on executive functions. This shift between the Bayley-II-NL and the Wechsler scales limits direct comparisons between 2 and 5 years. Despite the fluctuation in absolute scores, the significant association between cognition at 5 years and at school age could reflect the shared test structure, which assesses similar cognitive domains across both timepoints (20, 24–26). A possible explanation for the association between cognition at all ages, but specifically 2 and 8 years, despite the instrument shift, is that cognitive development may be less shaped by CHD specific factors than motor outcomes. Instead, it may be more strongly influenced by stable determinants such as parental cognitive ability and the home environment, consistent with the heritability of intelligence present in early childhood (35). Children with genetic syndromes known to directly influence cognition were excluded from this cohort, which may further explain why the observed pattern of cognitive development in our sample resembles that of typically developing children.

Children in our study did not show lower cognitive performance at 5 and 8 years compared to normative means. This contrasts with Naef et al. (16), and Sterken et al. (17), who reported that children with CHD perform below normative means. This discrepancy could reflect differences in cohort composition as both Naef, et al. (16) and Sterken, et al. (17) included larger, more heterogeneous CHD populations. Consistent with Naef et al. (16) and Sterken et al. (17), we did observe a mild improvement over time, especially for processing speed. However, a larger proportion of children in our study scored in the low average range compared to the expected norm. These distributional findings are consistent with findings by Latal, el al. (13). and Sterken, et al. (17) that the IQ of children with CHD is generally lowered by 5–10 IQ-points compared to healthy peers. A reduction of this size may seem small on group level, but is clinically relevant on an individual level, as it can have implications for a child's educational trajectory (36).

### Interplay between motor, cognitive and EF development

We did not find associations between motor, cognitive and EF development. This could partly be explained by the small sample size, but also by the limited range of EF functions assessed in this study. EF may underlie both motor and cognitive performance (14, 37), and in children with CHD, EF deficits adversely impact other developmental domains, as basic skills remain intact but their integration fails (9).

Li, et al. (38) propose that motor and cognition share neurodevelopmental pathways, and Liamlahi, et al. (39) found that impairments in any developmental domain indicate a broader neurodevelopmental vulnerability in children with CCHD. The absence of an association between these domains in our data may therefore reflect measurement limitations rather than an absence of interplay. The used NEPSY subtests in our study have limited working memory demands, which might be the specific EF component underlying the interplay between motor and cognitive development. Working memory tasks are consistently linked to co-activation of the cerebellum and dorsolateral prefrontal cortex. This circuit is implicated in both motor and cognitive development (40). This could explain that we did not find correlations between motor, cognition, and EF. Larger studies using EF measures with varying working memory demands are needed to clarify the interrelations between motor, cognition, EF and their neurobiological underpinnings. Prospective studies such as the ongoing trial on the effectiveness of a personalised EF-training program for preschool-aged children with CCHD (ClinicalTrials.gov NCT06267430), will also be informative in understanding whether targeted EF interventions improve later developmental outcomes.

### Motor and cognitive development in subgroups

Despite the use of different instruments across timepoints, we observed no improvement or deterioration over time in both the motor and cognitive domains. Two stable trajectories emerged from preschool to school age with one characterized by lower performance compared to test norms and the other by higher performance across all time points. Birth weight was significantly lower in the low-performance cognition group. This exploratory finding isconsistent with findings by Trivedi, et al., who reported lower birthweight as a predictor for poorer neurodevelopmental outcomes in children with CHD (41). Increased vulnerability to perioperative complications may partly explain this association (42). Other risk factors, such as diagnosis or surgical procedures, were not linked to neurodevelopmental impairments in the current study (43). Alternatively, brain immaturity and brain injury may contribute (43). The original RCT reported postoperative cerebral injury in 72%–78% of neonates, which indicates that these children have an increased risk of neurological impairment (4). The relation between perioperative brain injury and neurodevelopmental outcomes is beyond the scope of this descriptive study. However, it is the focus in ongoing work integrating perioperative neuroimaging with standardized developmental assessments (44, 45).

### Implications for developmental screening

Motor development appears to fluctuate from preschool to school age, although children seem to remain within their own relatively low or higher performing trajectory. This suggests that a single motor assessment may still signal whether a child belongs to a lower- or higher performing group, even without stable absolute scores over time. Our cognitive results showed that outcomes at 2 years may predict school-age outcomes, which underlines the importance of early cognitive screening in children with CCHD. However, the comprehensive test battery combining cognitive and EF measures was too lengthy for children and resulted in missing data. This highlights the need for shorter, more age-appropriate assessments. If genetic or medical risk factors are absent, a brief cognitive screening instrument at 2 years, such as the Ages and Stages questionnaire may be sufficient to determine if cognitive problems are present (46). This emphasizes the importance of drafting and using international guidelines for neurodevelopmental follow-up for children and adolescents with CCHD.

### Strengths and limitations

The generalizability of our findings is limited due to the small sample size and incomplete longitudinal data. Although no significant differences in neurodevelopmental outcomes were found between cardiac diagnoses or sexes, the small sample size also limits the power to detect such differences. The use of age-appropriate, validated instruments across three developmental stages is a strength, in light of the scarcity of longitudinal studies in this population. However, different measurement instruments across time points may limit direct comparability, complicating the interpretation of developmental trajectories. The selection of AARS and Inhibition for EF analysis was partly determined by data completeness, and these subtests do not capture all relevant EF domains at 8 years.

Although we extracted available information on paramedical interventions from patient medical records, these data were not systematically collected as part of the study protocol, and are therefore probably incomplete. This limits our ability to fully account for potential confounding effects of rehabilitation exposure on developmental trajectories and assessment performance. This should be acknowledged as a source of unmeasured heterogeneity in our longitudinal findings. Despite these limitations, this study in CCHD is among the few to examine multiple developmental domains from toddlerhood to school age, while also examining potential interactions across domains.

## Conclusion

In our study, children with CCHD showed moderately lower motor performances and comparable cognitive performance relative to normative means. Cognition measures were interrelated, whereas motor measures were not. Birthweight emerges as a possible risk factor for cognitive impairments. Future studies with larger samples should clarify potential relationships between motor, cognition, and EF and to specifically and specifically assess the role of EF in shaping motor and cognitive developmental trajectories. Understanding these bidirectional relationships is essential to improve early identification, tailored interventions, and optimize long-term neurodevelopmental outcomes in children with CCHD.

## Institutional review board statement

Although this study involves human participants, it was exempted from review under the Medical Research Involving Human Subjects Act (WMO). The Medical Ethics Committee of Utrecht University issued a non-WMO statement (ref. 13/442; September 5, 2013), confirming that this research does not fall within the scope of the WMO.

## Data Availability

The raw data supporting the conclusions of this article will be made available by the authors, without undue reservation.
