# Supplementary material for: From toddler to school age: longitudinal motor and cognitive outcomes in children with critical congenital heart disease
Source: Front Pediatr. 2026 Sep 23;14:1943055. doi: 10.3389/fped.2026.1943055 (PMC13642696; doi:10.3389/fped.2026.1943055)
Supplement: Supplementary file 2 [file Supplementaryfile2.docx]

**Supplement 2: Description of outcome measures**

*Motor outcome*

The Dutch version of the Bayley Scales of Infant Development, Third Edition (Bayley-III-NL) was used.^26^ The Bayley-III-NL is a standardised assessment tool to assess the development of children between 1 and 42 months of age in 5 domains: cognitive, language, motor, social-emotional, and behaviour scale. The Bayley-III motor scale consists of a separate score for fine motor skills (FM scaled score) and gross motor skills (GM scaled score). Both scaled scores are combined in the motor composite score. The Bayley-III-NL is a practical assessment tool to identify infants and toddlers with abnormal motor development. This tool allows the user to compare the gross and fine motor skills of infants and toddlers with normative data in order to discriminate between typical development and/or abnormal development. In addition, the tool can also be used to evaluate motor development between 0 and 42 months.

Motor composite scores (mean 100, SD 15) were used, and the separate FM scaled scores and GM scaled scores were analysed (mean 10, SD 3). Furthermore, classifications based on composite and scaled scores were applied. A motor composite score ≤ 70 or a fine or gross motor scaled score ≤ 4 (2 SD below the mean) indicates an *“abnormal motor score”* or “(*significant) motor delay*”. GM and FM scaled scores were categorised as ‘*below average*’ when motor scores were between -2 SD and ≤ –1 SD and *‘(above)* *average*’ when motor scores were > -1SD. The Bayley-III-NL classification system was used to classify motor development as “*average*” (motor composite score ≥ 90), “*below average*” (motor composite score ≤ 89) or “*delayed*” (motor composite score ≤ 79).

At 5 years, the Movement Assessment Battery for Children – Second Edition (Movement ABC-II-NL) was used to investigate motor performance and identify children with motor problems.^27^ The Movement ABC-II-NL is a global test of motor competence, with assessments of both fine and gross motor coordination. This test is divided into three different age bands: 3–6, 7–10, and 11–16 years. Each age band consists of eight items, which are divided into three subsets: manual dexterity, aiming and catching, and balance. Raw scores are converted into standard scores (1–19) and percentile scores (0–100). Scores above the 16th percentile are regarded as average motor performance. Scores between the 6th and 16th percentile are considered ‘at risk’ for motor difficulties, and scores below the 6th percentile indicate significant motor difficulties.

Motor development at 8 years was evaluated using the Bruininks–Oseretsky Test of Motor Proficiency, second edition (BOT-II-NL).^28^ This screening tool for children, aged 4 to 21, measures gross motor skills with five subtests and fine motor skills with three subtests. Items are scored on the performance of each task. The result of the test is an overall measure of motor proficiency, and additionally, scores are provided in four subscales: 1) fine manual control (FMC), 2) manual coordination (MC), 3) body coordination (BC) and, 4) strength/agility (SA). FMC encompasses motor skills involving control and coordination of the distal musculature of the hands and fingers; MC encompasses motor skills involving control and coordination of the arms and hands, especially for object manipulation; BC encompasses control and coordination of the large musculature used in maintaining posture and balance; and SA encompasses aspects of fitness and coordination involved in casual play, competitive sports, and other physical activity. Each motor composite includes 2 subtests. Scores for each subscale are reported as total point scores; scale scores (M = 15; SD = 5), standard scores (M = 50; SD = 10); or percentile ranks. Finally, the subscale composite standard scores are summed, and this sum is converted to a Total Motor Composite standard score (M = 50; SD = 10). In addition, findings can be reported as Descriptive Categories ranging from “*well-below average*” to “*well-above average*” scale scores (M = 15; SD = 5).

*- Cognitive outcome measures*

At 2-year follow-up, cognitive development was evaluated with the Dutch Bayley Scales of Infant and Toddler Development, Third Edition.^26^ This tool allows the user to compare different developmental domains of infants and toddlers with normative data to discriminate between typical and/or abnormal development. The cognitive domain of the Bayley-III-NL results in a cognitive composite score (mean 100, SD 15).

At 5-year follow-up cognitive development was evaluated with the Dutch Wechsler Preschool and Primary Scale of Intelligence (WPPSI-III-NL).^29^ It contains seven mandatory subtests for children 4:0 – 7:11 years of age, five supplementary subtests, and two optional subtests. The subtest scores are combined into a total intelligence quotient (TIQ), a performal intelligence quotient, a verbal intelligence quotient, and a processing speed index score (PS). The TIQ was used for this trial, and the PS index as well, since executive functions are involved in the associated subtests.^29^

At 8 years of age, the WISC-III-NL^30^ and the latest edition, WISC-V-NL^31^ were used to measure cognitive development. The WISC-IV-NL was used when this became available near the end of the study. The WISC-III-NL contains ten mandatory subtests and three supplementary subtests. The subtest scores are combined into a TIQ, PIQ, VIQ, and a PS index score. The TIQ and PS index were used for the current trial. The WISC-V-NL consists of ten mandatory subtests and five supplementary subtests. The subtest scores are combined into a TIQ, a verbal comprehension index, a visual spatial index, a fluid reasoning index, a working memory index, and a processing speed index. The TIQ and the PS index were used for this trial.

*Executive functioning measure*

Executive functioning was measured with subtests of the Developmental Neuropsychology Assessment, 2^nd^ edition (NEPSY-II-NL) for children 3:0 - 12:0 years of age.^32^ The selected subtests are derived from the Attention and Executive Functioning domain, and include the Auditory Attention and Response Set (ability to maintain selective and sustained auditory attention, and the ability to shift and maintain a new set), and Inhibition (ability to inhibit automatic responses, and the ability to switch between response types). A composite score was calculated, using American normative data, as Dutch scaled norm scores are not available for these subtests.
