# Supplementary material for: From toddler to school age: longitudinal motor and cognitive outcomes in children with critical congenital heart disease
Source: Front Pediatr. 2026 Sep 23;14:1943055. doi: 10.3389/fped.2026.1943055 (PMC13642696; doi:10.3389/fped.2026.1943055)
Supplement: Supplementary file 3 [file Supplementaryfile3.docx]

**Supplement 3: K-means longitudinal cluster analysis**

To identify trajectories of motor and cognitive development, k-means longitudinal clustering on the total motor respectively cognitive outcomes at age 2, 5, and 8 was performed in R using the ‘kml’ package. The kml package clusters individuals in groups based on the patterns of their individual trajectories.

Participants with outcomes missing at more than one timepoint were excluded (cognition n=4, motor n=5), and remaining missing data was imputed using the copy mean method as advised by the authors of the kml package (cognition n=14 imputations, motor n=10 imputations).

The optimal number of clusters was based on 20 iterations and determined using the Calinski/Harabasz criterion. Given the limited sample size, solutions with few clusters were preferred. For both motor and cognition data, the Calinski/Harabasz criterion clearly showed the highest values for a 2 cluster solution (see Figures below). High values indicate compact and separated clusters. A 3 cluster solution was also investigated, but in both motor and cognition trajectories this resulted in 2 clusters including the majority of participants and one very small non-distinctive cluster with n=6 participants for the motor data and n=4 participants for the cognition data.

Given the small sample size, exploratively investigating predictors for cluster membership separately was preferred over a multivariate model with all predictors at one, since the latter would inherently be overfitted.

**Figure 1.** Motor: Cluster solutions as suggested by 5 criteria.

**Figure 2**. Cognition: Cluster solutions as suggested by 5 criteria.

**Figure 3.** Motor: Partitions with same cluster number sorted according to the Calinski & Harabatz criterion, best first. Right: Chosen partition with mean trajectories.

**Figure 4.** Cognition : Partitions with same cluster number sorted according to the Calinski & Harabatz criterion, best first. Right: Chosen partition with mean trajectories.

The used R code from the kml package kan be found below.

**R code**

#Code for cognition data, identical code used for motor data

#Prepare data file

names(cogn)

traj_matrix1 <- as.matrix(cogn[2:4])

cldCOGN <- clusterLongData(

traj = traj_matrix1,

idAll = cogn$id,

time = c(1,2,3),

varNames = "Cognition"

)

#Imputation

cldCOGNimp <- imputation(cldCOGN,method="copyMean",lowerBound="globalMin",upperBound="globalMax")

#Cluster analysis

kml(cldCOGNimp,nbClusters=2:6,nbRedrawing=20,toPlot="none")

#Visualisation

plotAllCriterion(cldCOGNimp)

try(choice(cldCOGNimp))

#Add chosen clusters to data

cogn$clust <- getClusters(cldCOGNimp,2,asInteger = TRUE)

cogn$clust <- factor(3 - as.numeric(as.character(cogn$clust)))

#Calculate cluster means

clust1 <- cogn$clust

traj1 <- cldCOGNimp@traj

traj1 <- as.matrix(traj1)

storage.mode(traj1) <- "numeric"

centers1 <- rowsum(traj1, group = clust1) / as.vector(table(clust1))

centers1_df <- data.frame(cluster = rownames(centers1), centers1, row.names = NULL)

centers1_df

#Plot clusters

plot(cldCOGNimp,2,parTraj=parTRAJ(col="clusters"))
