# Supplementary material for: From toddler to school age: longitudinal motor and cognitive outcomes in children with critical congenital heart disease
Source: Front Pediatr. 2026 Sep 23;14:1943055. doi: 10.3389/fped.2026.1943055 (PMC13642696; doi:10.3389/fped.2026.1943055)
Supplement: Supplementary file 4 [file Supplementaryfile4.docx]

**Supplement 4: Correlation matrix with significant correlations after Holm-Bonferroni correction.**

|  | 1 | 2 | 3 | 4 | 5 | 6 | 7 | 8 | 9 | 10 | 11 | 12 | 13 | 14 |
| --- | --- | --- | --- | --- | --- | --- | --- | --- | --- | --- | --- | --- | --- | --- |
| 1. Bayley-III-NL: Total Motor | 1 |  |  |  |  |  |  |  |  |  |  |  |  |  |
| 2. Bayley-III-NL: Fine Motor | 0.83* | 1 |  |  |  |  |  |  |  |  |  |  |  |  |
| 3. Bayley-III-NL: Cognition score | 0.62* | 0.72* | 1 |  |  |  |  |  |  |  |  |  |  |  |
| 4. Bayley-III-NL: Gross motor |  | - | - | 1 |  |  |  |  |  |  |  |  |  |  |
| 5. MABC-II-NL: Total | - | - | - | - | 1 |  |  |  |  |  |  |  |  |  |
| 6. MABC-II-NL: Manual Dexterity | - | - | - | - | 0.84* | 1 |  |  |  |  |  |  |  |  |
| 7. WPPSI-III-NL: Total IQ | - | - | - | - | - | - | 1 |  |  |  |  |  |  |  |
| 8. WPPSI-III-NL: Processing speed | - | - | - | - | - | - |  | 1 |  |  |  |  |  |  |
| 9. BOT-II-NL: Total | - | - | - | - | - | - | - | - | 1 |  |  |  |  |  |
| 10. BOT-II-NL: Manual Coordination | - | - | - | - |  |  | - | - | 0.86* | 1 |  |  |  |  |
| 11. BOT-II-NL: Strength/Agility | - | - | - | - | - | - | - | - | 0.83* |  | 1 |  |  |  |
| 12. BOT-II-NL: Fine Manual Control | - | - | - | - | - | - | - | - | 0.68* | 0.68* | - | 1 |  |  |
| 13. BOT-II-NL: BC | - | - | - | - | - | - | - | - | 0.68* | - | - | - | 1 |  |
| 14. WISC-III-NL/WISC-V-NL: Total IQ | - |  | 0.71* | - | - | - | 0.82* | - | - | - | - | - | - | 1 |

*p < 0.001
