# Supplementary material for: From toddler to school age: longitudinal motor and cognitive outcomes in children with critical congenital heart disease
Source: Front Pediatr. 2026 Sep 23;14:1943055. doi: 10.3389/fped.2026.1943055 (PMC13642696; doi:10.3389/fped.2026.1943055)
Supplement: Supplementary file 5 [file Supplementaryfile5.docx]

**Supplement 5: Clusters in motor and cognitive outcomes across the 3 timepoints (T), resp 2, 5 and 8 years.**

Table 5a. Clusters in motor outcomes

| **Cluster** | **T1 (SD)** | **T2 (SD)** | **T3 (SD)** |
| --- | --- | --- | --- |
| 1 | 0.6 | 0.7 | 0.8 |
| 2 | -0.5 | -0.7 | -0.7 |

Table 5b. Clusters in cognitive outcomes

| **Cluster** | **T1 (SD)** | **T2 (SD)** | **T3 (SD)** |
| --- | --- | --- | --- |
| 1 | 1.1 | 1.1 | 0.9 |
| 2 | -0.6 | -0.6 | -0.7 |
